# Feasibility and acceptability of studying full-time nurse faculty salaries

**DOI:** 10.1186/s12912-024-02186-3

**Published:** 2024-07-29

**Authors:** Kathryn J. Malin, Jessica Zemlak, Jaqueline Christianson, Jessica Leiberg, Lisa Grabert

**Affiliations:** 1 College of Nursing, Marquette University, Milwaukee, USA; 2 School of Nursing, The University of Wisconsin Madison, Madison, USA

**Keywords:** Faculty, nursing, Workforce, Salaries and fringe benefits, Financial support, Schools, nursing, Health care economics

## Abstract

**Background:**

The nursing shortage is driven, in part, by the critical shortage of nursing faculty. Consequently, qualified potential nursing students are being turned away from nursing schools each year. The preeminent issue influencing the United States nurse faculty workforce shortage is salary; financial compensation is higher in clinical and private-sector settings than educational settings. The purpose of this study is to describe current full-time nurse faculty salary, sources of income, and perceived need for more income, as well as the feasibility and acceptability of research focused on full-time nurse faculty salaries, so to guide future nursing faculty workforce research.

**Methods:**

Using a cross-sectional study design, full-time nursing faculty working in a Midwestern state in the United States completed a survey inclusive of fixed choice and free text response options focused on income (individual gross, faculty, and secondary), demographics, and feasibility/acceptability of the survey instrument. Descriptive statistics were used to describe salary, sources of income, and perceived need for additional income. Feasibility and acceptability were evaluated by descriptive statistics examining three feasibility and acceptability questions, and by comparing demographic differences between participants who answered income questions compared to those who did not using *t* tests, *x*^*2*^ tests, and Wilcoxon signed rank tests.

**Results:**

One hundred and eighty-three full-time nurse faculty completed the survey in six weeks, representing 27% of the full-time nurse faculty workforce in the state. Over half of participants, 57%, reported having another job(s) in addition to their full-time faculty position to support basic living expenses. Most respondents reported willingness to share financial/salary data and viewed the survey to be acceptable.

**Conclusions:**

Research focused on nursing faculty salaries was feasible and acceptable to those who chose to participate in the study. Perceived income needs may be an important factor driving career decisions for nursing faculty. Future research should focus on delineating how salary influences the decision to enter or stay in the nursing faculty workforce. Further, this study can inform policy recommendations on how to best measure and report nurse faculty salary and the gap between clinical salaries and faculty salaries.

## Background

The nursing shortage has reached a crisis level. In 2022, hospitals reported a 17% vacancy rate and a 27% annual nurse turnover rate [1]. Nursing shortages and high nurse turnover rates directly contribute to missed nursing care, [2, 3] compromised nursing care, [4] decreased patient satisfaction [5], poor mental health [5], and increased cost to healthcare systems [5]. In the United States, 200,000 new nurses are needed by 2031 to replace nurses leaving the workforce [6]. Globally, the World Health Organization predicts a need for over 5 million nurses to address the nursing labor shortage [7].

Despite this need, there are insufficient numbers of new nurses graduating from prelicensure programs to meet this demand [8]. In the United States (U.S.), the nursing workforce shortage is driven by issues of both supply and demand. There is a growing demand for nurses in clinical settings however, there is an inadequate supply of nurse faculty needed to train qualified nursing school applicants [9–11]. These dual shortages are interwoven, and resolution of the nurse faculty shortage is an essential step to address the larger nursing workforce shortage.

The nurse faculty shortage in the U.S. is also worsening, with the highest ever recorded vacancy rate of 8.8% of nursing faculty positions left unoccupied in 2022 [12]. Consequently, many qualified applicants to nursing schools were denied admission. In 2021, over 90,000 qualified nursing school applicants were refused admission [13]. The shortage of nursing faculty is driven by multiple factors, including an aging workforce, lack of doctoral-prepared faculty, and salary gaps between nursing faculty and clinical nurses [14, 15]. Most U.S. full-time nursing faculty positions require a master’s degree or higher [13] however potential nurse faculty could earn $40,000 or more annually as a master’s-prepared nurse in clinical practice as compared to a full-time faculty role [10]. The ability for similarly educated nurses to earn larger salaries in clinical practice than academia is thought to be the pre-eminent issue driving the nursing faculty shortage in the United States (U.S). [16]Nationally, the reported median salary gap between nurse faculty and clinical registered nurse roles (requiring an associates or bachelor’s degree for entry into practice was $2,640 in 2022 (nurse faculty median annual income was $78,580; clinical registered nurse median annual income was $81,220) [17]. However, most nurse faculty are masters or doctorly prepared with clinical earning potential lagging clinical nursing roles. For example, when compared to advanced practice nurses (entry into practice is a master’s degree or doctorate) ($125,900) the salary gap increased to $47,320 [17, 18]. Furthermore, data regarding nursing faculty often comes from reports garnered by state and national organizations on behalf of deans and not directly from nursing faculty [17]. There is a need to understand how individual nurse faculty address and consider salary.

Details regarding if and how full-time nurse faculty compensate for this salary gap are unknown. Many individuals may be unwilling to participate in research focused on salary and income; it is not well-established if nurse faculty find research examining these constructs acceptable or if individual nurse faculty are comfortable self-reporting salary and income information. A necessary step toward understanding the role of salary and income in the nurse faculty shortage is to test the feasibility and acceptability of a survey instrument delivered in an online platform to full-time nurse faculty. Therefore, the purpose of this study was to examine the feasibility and acceptability of survey research among full-time nurse faculty with questions addressing personal salaries and secondary sources of income to guide future nursing faculty compensation research. We aimed to (1) describe self-reported full-time nurse faculty salary, sources of income, and perceived need for more income among current full-time nurse faculty in a Midwestern U.S. state, (2) evaluate feasibility of recruitment of full-time nurse faculty to complete an online survey focused on salary and income, and (3) measure acceptability of a survey on self-reported salary and income.

## Methods

### Research study design, setting, and ethics approval

Using a cross-sectional design, full-time nurse faculty in a Midwestern state completed one survey focused on salary and income. Ethical approval was obtained from Marquette University. Informed consent was obtained from participants of the study.

### Participants and recruitment

Full-time nurse faculty were recruited from Colleges/Schools of Nursing in the state of Wisconsin; our sampling frame for this study included 686 full-time nurse faculty [19]. Included participants were 18 or older and currently employed full-time as a nurse faculty member. We sent our study flyer via email to Deans of Colleges/Schools of Nursing and to professional networks and contacts of research team members over the study recruitment timeframe September-October 2023. Potential participants accessed a digital Qualtrics survey where they were screened for eligibility, and if eligible, provided informed consent to participate. Participants then completed an anonymous, one-time survey including quantitative, fixed choice responses and free text response options. Responses to each question were required, but participants could indicate “prefer not to respond” as a response choice. This approach was taken to assess acceptability of questions and limit missing responses. Upon completion of the survey, participants were given an option to be taken to a separate Qualtrics survey, unlinked to the anonymous study survey, to provide an email address for receipt of a $25 Amazon gift card to thank them for their time and participation.

### Measures

Descriptive statistics regarding age, race, gender, nurse faculty role, salary (nurse faculty role), income (across all occupations and within the household), and perceived need for income were obtained. Personal and household income questions were developed using previously published national surveys as a guide [20]. We asked participants to report income in three categories: gross, faculty, and secondary income and queried all three categories as both a categorical and continuous variable.

Feasibility and acceptability measures included three Likert style questions to assess comfort with survey items and willingness to participate in future surveys focused on salary. Salary questions were asked using both categorical and continuous options to assess acceptability and preference for responding to questions regarding income. Acceptability of measures was additionally assessed by identifying percentages of “prefer not to answer” responses to questions.

### Data analysis

Data analysis was performed using STATA version 18. We summarized sample characteristics and feasibility and acceptability measures with descriptive statistics such as mean, median, and percentages. An aim of our study was to understand feasibility and acceptability of our survey instrument among full-time nurse faculty. Therefore, exploring missing data responses to salary and income questions was an important component of our research. To examine our missing data, we performed statistical tests (*t* test, *x*^*2*^ test, and Wilcoxon signed rank test) to all survey items with a missing rate of 20% or higher, to determine if there was a significant difference in demographic variables between participants who answered the individual income dollar amount question compared to participants with missing data. Comparisons to public data regarding full time nurse faculty demographics [19] in the Midwestern state were made to evaluate feasibility of recruitment of a representative sample. For all statistical analysis, an alpha value of 0.1 was used.

## Results

### Demographics

Over a quarter of potentially eligible participants, in our targeted Midwestern state, completed our survey; 189 full-time nursing faculty started the survey, approximately 27% of the approximately 686 full-time nursing faculty in (blinded for review) were eligible to participate. [19] A total of six (3.2%) only answered the demographic questions and were excluded from the subsequent analysis. Participant demographics are reported in Table 1.

**Table 1:** Participant characteristics and feasibility measure (*n* = 183)

Response	Mean (SD/IQR)
Age	47.3 (10.4)
Years of Clinical Nursing Experience	11.0 (6.0, 18.0)
Years of Faculty Experience	8.0 (3.5, 13.0)
	**n (%)**
Race	
White	167 (92.8)
Other (including African American/Black, Asian, Native American/Alaskan Native, and Two or More Races)	9 (5.1)
Unknown/Not Reported	7 (2.1)
Sex	
Female	170 (94.4)
Male	8 (4.4)
Prefer Not to Answer	2 (1.1)
Faculty Income (*n* = 180)	
$0–40 K	2 (1.1)
$40–65 K	32 (17.8)
$65–80 K	60 (33.3)
$80–100 K	63 (35.0)
$100–150 K	21 (11.7)
$150–200 K	1 (0.6)
$200K+	1 (0.6)
Highest-Level Degree	
Master’s	109 (60.9)
Doctorate of Nursing Practice (DNP)	31 (17.3)
PhD	35 (19.6)
Other	4 (2.2)
Faculty Role	
Clinical/Non-Tenure Track	91 (52.3)
Tenured/Tenure Track	49 (28.2)
Other	34 (19.5)
Contract Length	
8–10 months	129 (72.1)
12 months	49 (27.4)
Other	1 (0.6)
Type of Institution	
Technical College	67 (37.4)
Community College	8 (5.0)
College or University	103 (57.5)
Public or Private Institution	
Public	115 (65)
Private	62 (35)
Type of Program Taught**	
Licensed Practical Nursing	24 (13.5)
Associate Degree in Nursing	75 (42.1)
Bachelor’s Degree in Nursing	79 (44.4)
Master’s Degree in Nursing	57 (32.0)
Doctorate in Nursing (DNP)	29 (16.3)
Doctorate in Nursing (PhD)	12 (6.7)
Other	9 (5.1)
Acceptability of Survey: How difficult was it for you to answer the survey questions?	
Extremely Difficult	0 (0)
Slightly Difficult	9 (5.1)
Neither Easy nor Difficult	47 (26.6)
Slightly Easy	33 (18.6)
Extremely Easy	88 (49.7)
Agreement with statement: I am willing to share financial information in an anonymous survey when the purpose is to study the nurse faculty shortage.	
Strongly Disagree	3 (1.7)
Disagree	6 (3.4)
Neither Agree nor Disagree	20 (11.5)
Agree	73 (42)
Strongly Agree	72 (41.4)

*Reponses for “other” included: PhD & DNP (1), EdD (1), currently working towards DNP (1), and finishing DNP currently (1)

**Indicates that participants may select more than one option, e.g., could indicate that they teach both at the bachelor’s and master’s level

### Salary and benefits

Over two thirds of respondents reported gross, and faculty salary between $65–100 K (Table 1). Over 75% of respondents with secondary income reported such income in the range of $1–30 K. Non-salary benefits of the full-time faculty role reported by respondents included health insurance, retirement benefits, job flexibility, and tuition remission for self-and/or family members.

### Secondary income characteristics

**Fig. 1:**
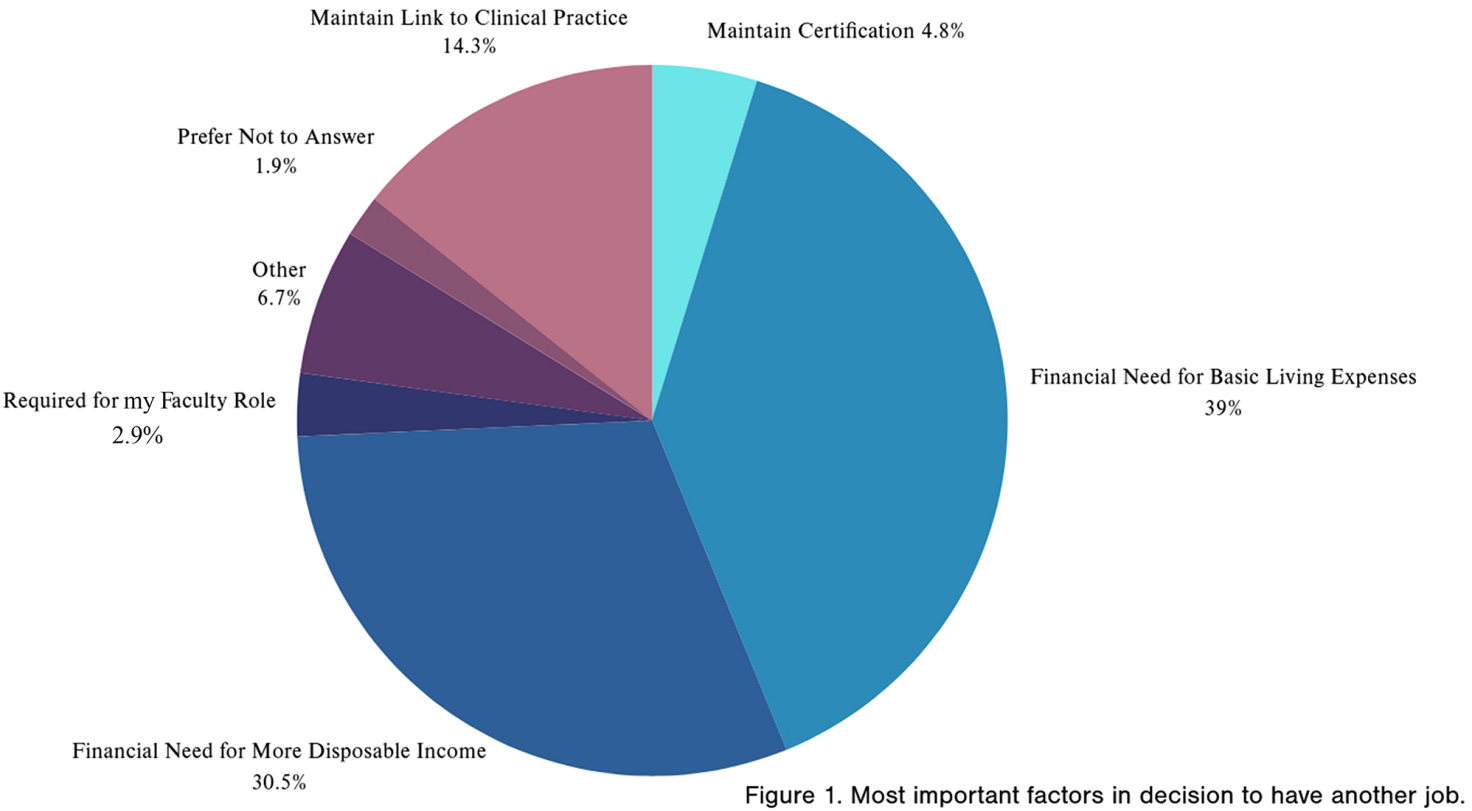
Most important factors in decision to have another job

Some participants perceived the need for additional income in addition to their full-time nurse faculty salary or took on additional job(s) to meet the perceived need for additional income. Most participants (*n* = 168, 89%) reported the perceived need to rely on other income to meet their basic needs. Over half, (*n* = 105, 57%) of participants reported having an additional job(s) or taking additional teaching responsibilities in addition to their contracted full-time nurse faculty position. Further, many participants (*n* = 109, 59%) stated they rely on other members of their household to meet their household income needs. The most important reasons for needing additional income are displayed in Fig. 1.

### Feasibility and acceptability

Most participants found the survey ‘slightly easy (n = 33, 17.5%) or ‘extremely easy’ (n = 88, 46.6%) to complete. A majority of participants, (n = 145, 76.7%) agreed they were willing to share financial information in an anonymous survey when the purpose was to study the nursing faculty shortage. Accordingly, nearly all participants (n = 181, 95.8%) provided a categorical range for their individual gross income and a majority of participants (n = 132, 69.8%) provided the dollar amount of their individual gross income in free text. Most participants indicated that they were ‘extremely likely’ (n = 96, 50.8%) or ‘likely’ (n = 63, 33.3%) to complete similar surveys about the nursing faculty shortage in the future. To ensure our sample reflected our broader sampling frame, we examined our demographic data and a state-wide data report collected from nursing education institutional administrators and found our sample demographics were similar. [19] Comparisons to public data regarding full time nurse faculty demographics [19] in the state of Wisconsin to that of participants in our study demonstrated a representative sample (Table 2).

**Table 2:** Sample characteristics and state nursing faculty characteristics

Characteristic	Sample (*N* = 183)	State of Wisconsin (as reported by Deans) [19] ^1^
*Race/Ethnicity*		
Other (including American Indian/Alaskan Native, Asian, Black, Hispanic, Hawaiian/Pacific Islander, Two or more races)	5.1%	9%
White	92.8%	85.0%
Unknown/Not Reported	2.1%	6.0%
*Sex*		
Female	94.4%	93.8%
Male	4.4%	6.2%
Unknown/Not Reported	1.2%	N/A
*Faculty Positions*		
Full-Time Tenure/Tenure Track	28.2%	33%
Full-Time Non-Tenure	52.3%	61%
Other/Prefer Not to Answer^2^	19.5%	N/A
*Highest Nursing Degree*		
Master’s in Nursing	60.9%	62.2%
Doctorate of Nursing Practice (DNP)	17.3%	13.7%
PhD in Nursing	19.6%	17.7%
Other/Prefer Not to Answer^3^	2.2%	N/A

1. State of Wisconsin

2. Other responses to Faculty Position in study sample included program chair, master teacher/AND, Instructional academic staff, performance level-based position

3. Other responses to Highest Nursing Degree included currently finishing degrees, EdD, and combined PhD/DNP.

**Fig. 2:**
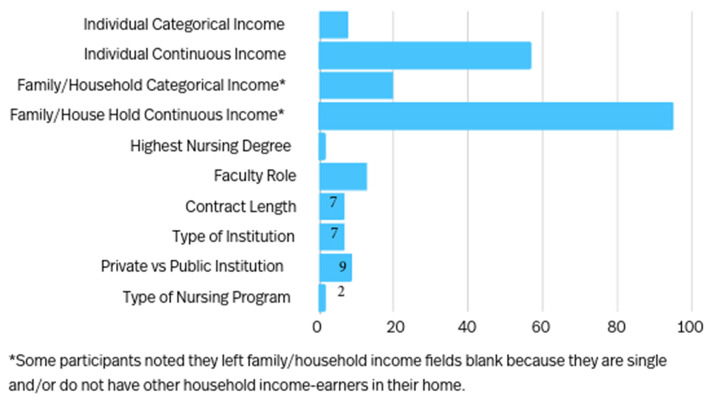
Frequency of missing data by variables (*n* = 189)

We asked similar questions examining salary and income from full-time jobs, family sources, and secondary jobs/sources of income. This approach was taken to examine if categorical versus free text, continuous responses to salary questions were more acceptable to participants. We wanted to examine if our missing data was missing at random or if there were significant patterns in our data which may indicate questions were acceptable to certain individuals in our study. Frequencies of missing data are displayed in Fig. 2. Our analysis comparing the group reporting data to the group with missing data included two sample t-tests, Wilcoxon rank-sum, and Fisher’s exact tests. We found a statistically significant difference for type of institution (technical, community, or college/university), institution tax basis (private or public), and teaching in an associate nursing program for both faculty and secondary income as a continuous variable (Tables 3 and 4), representing possible sources of bias. Years of faculty experience was also statistically significant (with a positive association) as a difference for secondary income reporting (Table 4).

**Table 3:** Acceptability of reporting faculty income data among full-time nurse faculty (represented by continuous variable)

Factor	No missing data		Missing data		*p*-value
Faculty income (continuous)	121		62		
Age, mean (SD)^1^	46.7 (10.4)		48.7		0.21
Years of Clinical Nursing^2^ Experience	11.0 (6.0, 16.0)		11.0 (7.0, 19.0)		0.73
Years of Faculty Experience^2^	7.0 (4.0, 11.0)		8.0 (4.0, 15.0)		0.35
*Race* ^3^					0.51
White	114 (94.2)		56 (90.3)		
Black	2 (1.7)		3 (4.8)		
Native American/Alaskan Native	0 (0)		1 (1.6)		
Asian	1 (0.8)		1 (1.6)		
Two or more races	1 (0.8)		0 (0)		
Prefer not to answer	3 (2.5)		1 (1.6)		
*Ethnicity* ^3^					0.55
Non-Hispanic/Latino	115 (95.0)		57 (91.9)		
Hispanic/Latino	1 (0.8)		0 (0)		
Prefer not to answer	5 (4.1)		5 (8.1)		
*Gender* ^3^					0.87
Male	5 (4.1)		3 (4.8)		
Female	114 (94.2)		59 (95.2)		
Prefer not to answer	2 (1.7)		0 (0)		
*Gross Income Category* ^3^					0.24
$40–65 K	19 (15.7)		5 (8.3)		
$65–80 K	37 (30.6)		26 (43.3)		
$80–100 K	43 (35.5)		18 (30.0)		
$100–150 K	21 (17.4)		9 (15.0)		
$150–200 K	0 (0)		1 (1.7)		
$200K+	1 (0.8)		1 (1.7)		
*Faculty Income Category* ^3^					0.19
$0–40 K	1 (0.8)		1(1.7)		
$40–65 K	24 (20.0)		8 (13.3)		
$65–80 K	35 (29.2)		25 (41.7)		
$80–100 K	45 (37.5)		18 (30.0)		
$100–150 K	15 (12.5)		6 (10.0)		
$150–200 K	0 (0)		1 (1.7)		
$200K+	0 (0)		1 (1.7)		
*Highest Nursing Degree* ^3^					0.57
Master’s in Nursing	76 (63.30		34 (54.8)		
Doctorate in Nursing	21 (17.5)		16 (25.8)		
Doctorate in Nursing	20 (16.7)		11 (17.7)		
Other	3 (2.5)		1 (1.6)		
*Faculty Role* ^3^					0.20
Clinical/Non-Tenure Track	61 (53.0)		30 (49.2)		
Tenured/Tenure Track	28 (24.3)		22 (36.1)		
Other	26 (22.6)		9 (14.8)		
*Contract Length* ^3^					0.040*
8–10 months	93 (76.9)		38 (62.3)		
12 months	28 (23.1)		22 (36.1)		
Other	0 (0)		1 (1.6)		
*Type of Institution* ^3^					0.031*
Technical College	46 (38.0)		21 (34.4)		
Community College	10 (8.3)		0 (0)		
College or University	65 (53.7)		40 (65.6)		
*Institution Tax Basis* ^3^					0.071*
Public	36 (30.3)		27 (44.3)		
Private	83 (69.7)		34 (55.7)		
*Type of Program Taught* ^3^	*No*	*Yes*	*No*	*Yes*	
Licensed Practical Nursing	106 (87.6)	15 (12.4)	51 (85.0)	9 (15.0)	0.65
Associate in Nursing	63 (52.1)	58 (47.9)	42 (70.0)	18 (30.0)	0.025*
Bachelor’s in Nursing	71 (58.7)	50 (41.3)	30 (50.0)	30 (50.0)	0.34
Master’s in Nursing	82 (67.8)	39 (32.2)	41 (68.3)	19 (31.7)	1.00
Doctorate in Nursing	103 (85.1)	18 (14.9)	48 (80.0)	12 (20.0)	0.40
Doctorate in Nursing	114 (94.2)	7 (5.8)	55 (91.7)	5 (8.3)	0.54
Other	115 (95.0)	6 (5.0)	57 (95.0)	3 (5.0)	1.00

^1^ Two sample t-test

^2^ Wilcoxon rank-sum

^3^ Fisher’s exact

*Indicates significance at < 0.10

**Table 4:** Acceptability of reporting secondary income data among full-time nurse faculty (represented by continuous variable)

Factor	No missing data		Missing data		*p*-value
Secondary income (continuous)	65		40		
Age, mean (SD)^1^	47.7 (10.5)		45.4 (10.1)		0.27
Years of Clinical Nursing^2^ Experience	12.0 (7.0, 18.0)		11.0 (7.0, 17.5)		0.79
Years of Faculty Experience^2^	9.0 (4.0, 15.0)		5.0 (2.0, 9.5)		0.007*
*Race* ^3^					1.00
White	60 (92.0)		38 (95.0)		
Black	2 (3.0)		1 (3.0)		
Native American/Alaskan Native	0 (0)		0 (0)		
Asian	0 (0)		0 (0)		
Two or more races	0 (0)		0 (0)		
Prefer not to answer	3 (x)		1 (x)		
*Ethnicity* ^3^					0.50
Non-Hispanic/Latino	59 (91.0)		39 (98.0)		
Hispanic/Latino	1 (2.0)		0 (0)		
Prefer not to answer	5 (8.0)		1 (3.0)		
*Gender* ^3^					1.0
Male	2 (3.0)		1 (3.0)		
Female	62 (95.0)		39 (98.0)		
Prefer not to answer	1 (2.0)		0 (0)		
*Gross Income Category* ^3^					0.23
$40–65 K	12 (18.0)		3 (8.0)		
$65–80 K	20 (31.0)		18 (45.0)		
$80–100 K	19 (29.0)		13 (33.0)		
$100–150 K	13 (20.0)		5 (13.0)		
$150–200 K	0 (0)		1 (3.0)		
$200K+	1 (2.0)		0 (0)		
*Faculty Income Category* ^3^					0.39
$0–40 K	1 (2.0)		0 (0)		
$40–65 K	16 (25.0)		6 (15.0)		
$65–80 K	18 (28.0)		18 (45.0)		
$80–100 K	21 (32.0)		12 (30.0)		
$100–150 K	9 (14.0)		4 (10.0)		
$150–200 K	9 (14.0)		4 (10.0)		
$200K+	0 (0)		0 (0)		
*Highest Nursing Degree* ^3^					0.39
Master’s in Nursing	41 (64.0)		20 (50.0)		
Doctorate in Nursing	11 (17.0)		7 (18.0)		
Doctorate in Nursing	11 (17.0)		12 (30.0)		
Other	1 (12.0)		1 (3.0)		
*Faculty Role* ^3^					0.83
Clinical/Non-Tenure Track	35 (56.0)		20 (50.0)		
Tenured/Tenure Track	14 (23.0)		10 (25.0)		
Other	13 (21.0)		10 (25.0)		
*Contract Length* ^3^					0.26
8–10 months	53 (82.0)		29 (73.0)		
12 months	12 (18.0)		10 (25.0)		
Other	0 (0)		1 (3.0)		
*Type of Institution* ^3^					0.056*
Technical College	24 (37.0)		13 (33.0)		
Community College	7 (11.0)		0 (0)		
College or University	34 (52.0)		27 (68.0)		
*Institution Tax Basis* ^3^					0.14
Public	19 (30.0)		18 (45.0)		
Private	x (x)		x (x)		
*Type of Program Taught* ^3^	*No*	*Yes*	*No*	*Yes*	
Licensed Practical Nursing	56 (86.0)	9 (14.0)	34 (87.0)	5 (13.0)	1.00
Associate in Nursing	33 (51.0)	32 (49.0)	28 (72.0)	11 (28.0)	0.041*
Bachelor’s in Nursing	34 (52.0)	31 (48.0)	21 (54.0)	18 (46.0)	1.00
Master’s in Nursing	45 (69.0)	20 (31.0)	22 (56.0)	17 (44.0)	0.21
Doctorate in Nursing	58 (89.0)	7 (11.0)	31 (79.0)	8 (21.0)	0.5
Doctorate in Nursing	60 (92.0)	5 (8.0)	38 (97.0)	1 (3.0)	0.41
Other	63 (97.0)	2 (3.0)	37 (95.0)	2 (5.0)	0.63

^1^ Two sample t-test

^2^ Wilcoxon rank-sum

^3^ Fisher’s exact

*indicates significance at < 0.10

## Discussion

Our results add valuable information regarding the state of the science examining nurse faculty compensation and the feasibility of directly asking nurse faculty about salary and compensation through survey research. Many participants identified the need to supplement their incomes to meet basic needs. This compensation issue aligns with similar research among faculty which identified low compensation as foremost reason for considering leaving academia [15]. We found our survey method and instrument exploring salary and compensation to be feasible and acceptable to nurse faculty, laying critical groundwork for future, larger national research addressing similar topics.

### U.S. nursing faculty income

It is not surprising nurse faculty perceive the need for additional income. Our study found that nurse faculty supplement their faculty income with additional job(s) (57%) and rely on income from other household members to meet basic needs (59%). Current salary trends in academic and clinical settings indicate the issue around salary gaps is worsening. For example, the salary gaps between clinical nursing to nursing faculty and advanced practice nurse to nursing faculty both increased from 2021 to 2022 due to nurse faculty wage stagnation. Nurse faculty wages increased only 1.4% from 2021 to 2022, whereas clinical registered nurse salaries increased by 4.7% and advanced practice nurse salaries increased by 1.7% at the same time. [17, 18] There is a need to address the expanding gap between clinical and academic salaries with solutions necessary beyond individual academic institution level salary adjustments.

### Feasibility and acceptability of surveying nurse faculty on income

Our study is unique in that it surveyed nurse faculty directly. Previous studies of nurse faculty tend to capture indirect responses that are provided to national and state reporting entities by Deans (on behalf of faculty) of nursing colleges and universities. [19] We found completing an online survey examining sensitive information regarding salary was feasible and acceptable to our participants. The feasibility and acceptability we identified in our research of directly querying nurse faculty regarding compensation should be considered in future research.

### Policy and nurse faculty salaries

Our findings are consistent with the evidence supporting current policy efforts at the Federal level. These current proposed policies seek to address the salary gap between nurse faculty and the potential income faculty could earn compared to the clinical setting [21–24]. Specifically, H.R. 7002/S. 2815 (from the 118th Congress) [23] would provide funding to minimize the salary gap between the average nurse faculty salary and the average salary of nurses in clinical practice (H.R. 7002 2024 & S. 2815 2023) [25, 26] These bills would provide much needed transparency and clear data regarding nurse salaries. These bills would derive an estimate of the average nurse faculty salary for a given institution (via an attestation) of the average nursing faculty salary during the most recent 3-years, as submitted by participating schools of nursing. In addition, the bill is reliant on reporting of the salary history of nursing faculty if such faculty were in clinical practice for the most recent prior 3-year period (H.R. 7002 & S. 2815). If a school of nursing is unable (due to a small number of participants, etc.) to provide such information about prior employment, the Secretary of Health and Human Services has the authority to derive the information based on the “average local salary of nurses in clinical practice” (H.R. 7002 2024 & S. 2815 2023).

If such legislation were enacted, our survey provides valuable information about how such income information could be collected. Given the findings from our study, schools of nursing may also encounter data collection issues regarding continuous salary data for their employees for their previous clinical practice income. Our results suggest that any methods used to derive an average or median salary for nurse faculty could be biased if not controlled for institution type (technical, community, or college/university), institution tax basis (public or private), and teaching in an associate nursing program. The proposed legislation gives permissive authority to control for credentials, experience, and levels of education—none of which address the three factors that we found evidence of bias on observable characteristics.

### Implications for future research

Our results provide insight into factors that should be considered if academic institutions have a stated goal to increase diversity, equity, and inclusion among nurse faculty.

Nurse faculty roles are largely occupied by people who identify their gender as women and their race as white. These demographic factors may underpin larger gendered and racial privilege factors driving salary disparities among full-time nurse faculty. Women in the United States earn 82 cents for every dollar earned by their male counterparts for the same job [27]. Black/African American women earn 65 cents for every dollar earned by White men [27]. As the sample of people who identify as a race other than white and genders other than female was low both in our data, state, and national data of nurse faculty, it is difficult to understand how the intersecting factors of race and gender may contribute to nurse faculty shortages related to compensation. It is important to consider that groups already facing a pay disparity related to implicit bias in salary in the United States may be unwilling or unable to accept the pay disparity when moving from clinical to academic roles. More study is needed to understand the role of clinical and academic pay disparity on the achieving a diverse nursing workforce. Such research would inform retention efforts to keep current nurse faculty in the workforce but may also inform barriers to entry pertaining to recruitment of a future diverse nurse workforce.

### Limitations

Limitations of our study require consideration. We asked questions in multiple different ways regarding individual gross income, faculty income, secondary income, and household income, which may have contributed to response confusion among some participants. Our study included a sample in one state of the United States with only one fourth of possible participants’ responses, limiting generalizability outside of our sampling frame. Future research may benefit from broader geographic sampling and sample sizes sufficient to stratify data by academic institution type, program type, seniority, or type of nursing academic position. Further, it is possible that the respondents in our study were more likely to have salary concerns and thus more likely to complete the survey. Finally, our results do not include consideration for academic rank, type of institution, or type of nursing program and is problematic. These variables may have influenced our results and should be considered in future research.

As noted above, we had a missing data rate of over 20% for our continuous income variables. Because of this high missing rate, we have opted to suppress reporting of the mean for gross, faculty, and secondary income. Further, we are concerned about the accuracy of the income-related means because we have evidence of selection bias on several observable characteristics. Participant confusion regarding reporting the same variable twice may have also contributed to the high missing data rate for continuous income reporting. Additionally, data on what types of secondary income sources nurse faculty engage in to supplement their income such as number of jobs, job settings, and number of hours worked on average in supplemental jobs was not collected and may warrant future research.

## Conclusion

Alleviation of the nursing workforce shortage will only occur once the nursing faculty shortage is also resolved. Research focused on nurse faculty salaries and perceived needs for income is both feasible and acceptable. Our results indicate that many full-time nursing faculty perceive a need for more income to meet their financial needs. To meet these needs, they either take on additional job(s) and/or rely on other household income. This unsustainable financial reality may impact nurses’ decisions on types of employment and may have broader implications on the diversity of the nursing faculty workforce. Further, this preliminary work examined the financial realities many full-time nursing faculty face underscoring broader issues such as the possibility that economic privilege may be a baseline requirement to enter the nursing faculty role. Several unobservable characteristics that may impact salary, such as number of individuals in the household and number of dependents must also be studied.

Our research has implications for policies supporting a robust, diverse nurse faculty workforce. We identified that nurse faculty are supplementing their income to meet basic needs. This salary deficit coupled with the critical shortage of nurse faculty indicates a need for action. Universities and academic institutions alone cannot be charged with implementing the salary changes needed to address the nurse faculty shortage. Policies, such as H.R. 7002/S. 2815, which support funding and growth of nurse faculty salaries may have positive impacts on the nursing shortage.

## Data Availability

The datasets used and/or analyzed during the current study are available from the corresponding author on reasonable request.
